# Synthesis of highly monodisperse Pd nanoparticles using a binary surfactant combination and sodium oleate as a reductant†

**DOI:** 10.1039/d1na00052g

**Published:** 2021-03-05

**Authors:** Anna Pekkari, Xin Wen, Jessica Orrego-Hernández, Robson Rosa da Silva, Shun Kondo, Eva Olsson, Hanna Härelind, Kasper Moth-Poulsen

**Affiliations:** Applied Chemistry, Department of Chemistry and Chemical Engineering, Chalmers University of Technology 41296 Gothenburg Sweden mkasper@chalmers.se; Nano and Biophysics, Department of Physics, Chalmers University of Technology 41296 Gothenburg Sweden

## Abstract

This study presents the synthesis of monodisperse Pd nanoparticles (NPs) stabilized by sodium oleate (NaOL) and hexadecyltrimethylammonium chloride (CTAC). The synthesis was conducted without traditional reductants and Pd-precursors are reduced by NaOL. It was confirmed that the alkyl double bond in NaOL is not the only explanation for the reduction of Pd-precursors since Pd NPs could be synthesized with CTAC and the saturated fatty acid sodium stearate (NaST). A quantitative evaluation of the reduction kinetics using UV-Vis spectroscopy shows that Pd NPs synthesized with both stabilizer combinations follow pseudo first-order reaction kinetics, where NaOL provides a faster and more effective reduction of Pd-precursors. The colloidal stabilization of the NP surface by CTAC and NaOL is confirmed by Fourier transform infrared (FTIR) and nuclear magnetic resonance (NMR) analysis.

## Abstract 2

This study presents the synthesis of monodisperse Pd nanoparticles (NPs) stabilized by sodium oleate (NaOL) and hexadecyltrimethylammonium chloride (CTAC).

## Graphical abstract

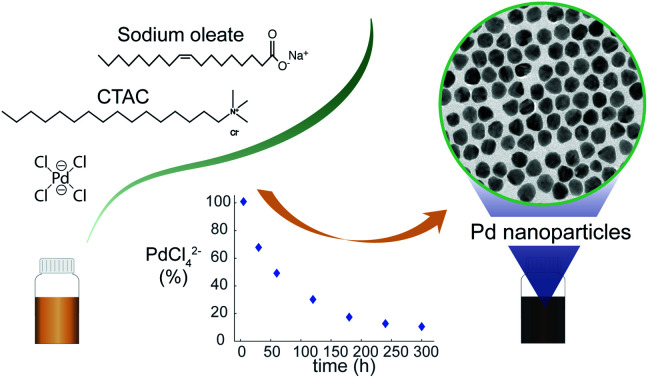

## 1 Introduction

In the last few decades, palladium (Pd) nanoparticles (NPs) have been intensively studied because of their unique properties rendering applications in fuel cell technology, hydrogen sensing and catalysis,^1–3^ and biomedical applications.^4^ Pd NPs are widely used as a catalyst for cross-coupling reactions of *e.g.* aryl halides with arylboronic acids (Suzuki coupling reactions) and with alkenes (Heck reaction). In the automotive industry, palladium is the major catalyst used for the reduction of vehicle exhausts. Pd NPs also show large capacity for hydrogen adsorption and have been used to fabricate hydrogen gas optical sensors by monitoring slight changes in the localized plasmon resonance peak (LSPR).^5^ As the properties of the NPs are influenced by the crystal structure and size, much effort has been invested into developing their synthesis procedures. The fine control of the size and shape of Pd NPs in aqueous media through facile synthetic methods is challenging due to the increase of cohesive energy as a function of the nanoparticle size.^6^ Among the synthesis methods, the solution-based colloidal method is one of the most widely explored ones because of its excellent control of the NP shape and size.^7,8^ By precise control of the reaction parameters, *e.g.* the precursor concentration, temperature, and capping agents, monodisperse NPs with defined shapes can be formed, and the properties can be tuned to optimize the performance in the desired application.^9–12^ For instance, monodisperse Pd NPs can restrain the increasing relationship between multiple intermediates on a catalyst surface. In the case of sensors, a more defined LSPR peak of monodisperse Pd NPs can leverage the design of more sensitive optical sensors.

The general procedure to synthesize NPs by the colloidal method involves the reduction of a metal precursor using a reductant. A variety of compounds have been employed as reductants in synthesis of Pd NPs including ascorbic acid, citric acid, and polyalcohols.^13–15^ The physicochemical properties of the reductant influence the reaction kinetics and thus the nucleation and growth of the Pd NPs.^15^ Therefore, careful selection of the reductant is important to control the quality of the NPs. Apart from donating electrons, the reductant can sometimes have several roles *e.g.* as a solvent, capping agent or colloidal stabilizer.^14^ Capping agents adsorb to the surface of the NPs and can provide colloidal stabilization. Certain stabilizers selectively adsorb onto the crystal facets of the NPs, and growth can then be directed into specific shapes.^9,11,16^

Among the different stabilizers employed in metal NP synthesis, fatty acids have been widely applied because of their high affinity to metals. In the synthesis of various metal NPs typical examples include lauric acid,^17–20^ palmitic acid,^17,21^ and stearic acid.^17,22,23^ The most frequently applied fatty acid capping agent in metal NP synthesis is the unsaturated fatty acid oleic acid (OA), and its sodium salt form, sodium oleate (NaOL). These fatty acids have been used as precursors or stabilizers in the synthesis of iron oxide NPs,^24–26^ Ag NPs,^27,28^ Pt NPs,^28–30^ and Cu(i) sulfide NPs.^31^

Apart from acting as a colloidal stabilizer, there have been indications that NaOL can partly reduce metal precursors when combined with the cationic surfactant hexadecyltrimethylammonium bromide (CTAB). Because of the shape-directing properties, this binary surfactant mixture has been applied in the synthesis of Pd nanodendrites^32^ and Au nanorods.^33,34^ In the growth of Au nanorods, NaOL was ascribed to contribute to the partial reduction of Au(iii) to Au(i) species by noticing a color change of the reaction solution.^33^ The reduction was attributed to the electron dense double bond in the alkyl chain of NaOL, which acts as an electron donating group to partly reduce Au(iii) species.

However, to our knowledge, investigation of the reduction of metal ions by the fatty acid NaOL has not been explored beyond Au, and only with the presence of traditional reductants such as ascorbic acid. The application of NaOL as a single reductant in the synthesis of uniform metal NPs may further simplify the method. Furthermore, an explanation of the reduction mechanism is currently lacking. There is no indication that the alkyl double bond is the sole contributor to reduction since the application of a saturated fatty acid as a reductant has not been studied.

In this study, we aim to synthesize uniform Pd NPs stabilized with NaOL and hexadecyltrimethylammonium chloride (CTAC), in the absence of traditional reductants in a simple one-pot approach. Different reaction conditions are explored to study the influence on Pd NP properties. This includes different reaction times, and temperatures, and evaluation of applying a single stabilizer. Investigation of the involvement of the alkyl double bond in the reduction of Pd-precursors is performed by replacement of NaOL with the saturated fatty acid sodium stearate (NaST). A quantitative evaluation of the reduction kinetics involved in the Pd NP synthesis is performed using UV-Vis spectroscopy. To understand colloidal stabilization, careful chemical evaluation of the Pd NPs is performed.

## 2 Results and discussion

### 2.1 Synthesis of Pd NPs stabilized with NaOL and CTAC and evaluation of reaction conditions

**Fig. 1:**
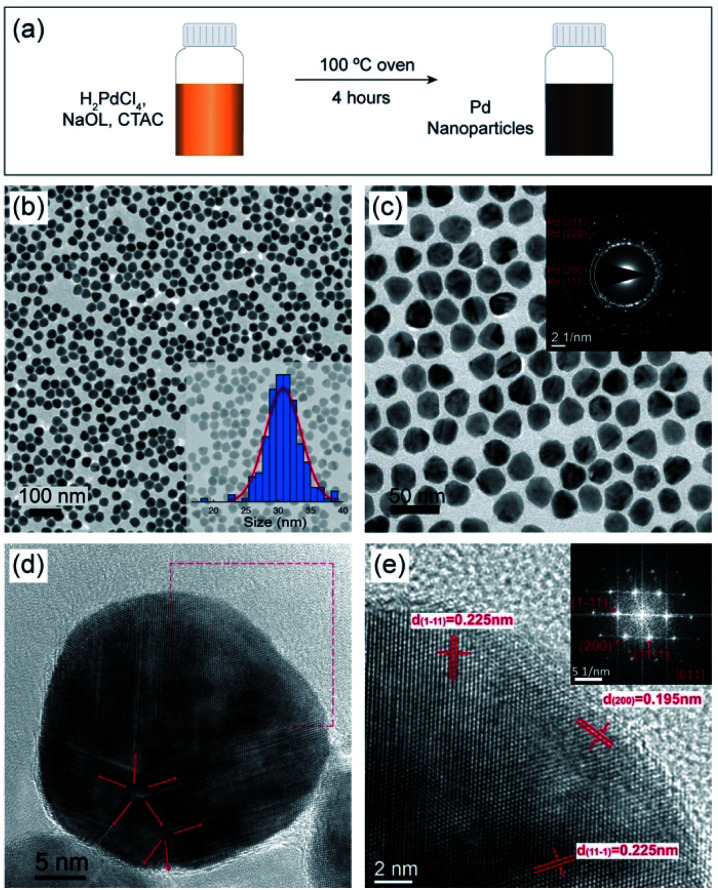
Characterization of Pd NPs stabilized with NaOL and CTAC. (a) Scheme of the Pd NP synthesis, (b) and (c) TEM images of monodisperse Pd NPs at relatively low magnification, where the inset in (b) shows the histogram of the particle size distribution with an average size of 29.7 nm ± 5.7%, the inset in (c) shows the SAED pattern of Pd NPs, in which diffraction rings correspond to (111), (200), (220) and (311) lattice planes of face-centered cubic Pd crystals. (d) HRTEM image of an individual Pd NP shows a multiple-twinned structure, where twin boundaries are marked with red arrows, (e) HRTEM image of the selected area of the Pd NP marked by a red square in (d), marking lattice fringes assigned to the {111} and {200} lattice planes having interplanar spacings of 0.225 nm and 0.195 nm respectively. The inserted image in (e) shows a corresponding FFT pattern indicating that the zone axis of this crystal unit is [011].

Uniform NaOL and CTAC-stabilized Pd NPs were synthesized without the use of traditional reducing agents using a simple one-pot approach (Fig. 1a). Fig. 1b and c show the TEM images of the highly monodisperse Pd NPs under two different magnifications, with an average size of 29.7 nm ± 5.7%, seen in the histogram of the size distribution (inset in Fig. 1b). As shown in the selected area electron diffraction (SAED) pattern (inset Fig. 1c), the formed Pd NPs indeed consist of Pd crystals, with diffraction rings corresponding to the (111), (200), (220) and (311) lattice planes of face-centered cubic Pd crystals. A further observation of the contrast on the Pd NPs in Fig. 1b and c shows that these Pd NPs consist of a mixture of single-crystal NPs (Fig. S1†) and multiple twinned structured NPs. A high-resolution transmission electron microscopy (HRTEM) image of an individual multi-crystalline Pd NP is shown in Fig. 1d. The twin boundaries radiate from two centers and are marked by red arrows. Fig. 1e displays a higher-magnified HRTEM of the crystal unit, marked by a red square in Fig. 1d. The lattice spacings of 0.225 nm and 0.195 nm on the surface of the NP crystal (marked in the figure) are indexed to the {111} and {200} Pd lattice planes. The corresponding Fast Fourier Transform (FFT) pattern indicates that the zone axis of this crystal unit is [011].

The synthesis of Pd NPs was performed under different reaction conditions to evaluate the influence on the particle size and shape. When the reaction was run for 1 h Pd NPs form but multiple particle seeds are present (Fig. S2a†). The number of seeds decreases after 2 h reaction time (Fig. S2b†), and almost none are present after 4 h (Fig. 1b and c). At a lower reaction temperature (50 °C), the orange Pd-complex remained insoluble and no reduction occurred (Fig. S3a†), whereas at 80 °C the Pd NPs have similar shapes to those synthesized under the optimal conditions (100 °C) (Fig. S3b†). Additionally, the robustness of the method was demonstrated when the synthesis was conducted in an oil bath for 2 h and 4 h, respectively, which produced equivalent Pd NPs (Fig. S4†).

Substitution of CTAC with CTAB, the equivalent ammonium salt with bromide as a counter ion, in the surfactant mixture favors the formation of Pd cubes, bars and “nano arrows” (Fig. S5†). Bromide ions (Br^−^) present in CTAB are well-known capping agents that selectively adsorb on the (100) crystal facets. This has led to the application of CTAB in the synthesis of Pd cubes^35,36^ and rods.^37^ After 4 h of reaction, the Pd NPs are smaller and multiple seeds are present compared to the synthesis with NaOL and CTAC. Despite using the same reductant, NaOL, the reduction was slower with CTAB, which was observed by a slower color change of the reaction solution. This indicates that fewer atoms can be reduced at any time point, explained by the stronger complexation between Pd-precursors and bromide ions in CTAB (PdBr_4_^2−^).

Investigations of the stabilizers NaOL and CTAC were performed to understand their roles in the reduction of the Pd-precursor. In order to evaluate the hypothesis proposed by previous studies that the reduction of the Pd-precursor is caused by the alkyl double bond in NaOL, NaOL was replaced by the equivalent saturated fatty acid NaST in the synthesis of Pd NPs. Interestingly, the synthesis with NaST and CTAC yielded Pd NPs with a size of 13 nm ± 19% (Fig. S6†). The reduction by NaST was slower which was observed by a later color change of the reaction solution. Despite NaST containing no double bond the reduction of the Pd-precursor occurred, which indicates that the reaction mechanism is more complex than the hypothesis proposed by previous studies.

Synthesis experiments were carried out to study the individual roles of the Pd-precursor, CTAC and NaOL/NaST in the synthesis. Synthesis conducted with only Pd-precursors results in no reduction which excludes the possibility that the Pd-precursor has reduction ability by itself. Furthermore, synthesis with only the Pd-precursor and CTAC gave no visible color change of the reaction solution after 4 h of reaction. Hence, under the present reaction conditions, CTAC alone could not reduce the Pd-precursor, and the reductant NaOL or NaST was necessary for the reduction of the Pd-precursor and the formation of Pd NPs. Moreover, Pd NPs stabilized with only NaOL or NaST were synthesized. NaOL-stabilized NPs (Fig. S7†) had poor colloidal stability in the original concentration of NaOL (8 mM) and at a higher concentration (15.9 mM). When the concentration of NaOL was further increased (39.8 mM), the colloidal stability slightly improved, and small NP seeds can be seen (Fig. S7b†). Furthermore, Pd NPs synthesized with only NaST as the stabilizer (Fig. S8†) resulted in a black precipitate that consisted of Pd NPs (Fig. S8c†). It is evident that both stabilizers, *i.e.* NaOL and NaST, could reduce the Pd(ii) precursor to form Pd NPs but could not form stable Pd NP suspensions. Hence, the combination with CTAC was necessary to provide sufficient colloidal stability.

CTAC is a cationic surfactant and used in the synthesis of several metal nanoparticles such as single-crystal gold nanospheres, and gold nanorods. It is mainly seen as a stabilizer or capping agent with a role of preventing the agglomeration of nanoparticles. CTAC also reacts with PdCl_4_^2−^ to form complex-surfactant postmicellar/organic salt aggregates.^38^ Upon the addition of CTAC/NaOL to Na_2_PdCl_4_, the colour of the solution changed from yellow to orange and then it transformed into a orange precipitate. Kaur *et al.* showed that the precipitation of Pd(ii) and CTAC could be due to metalomicelles in water.^38^ This is also observed for Pd-CTAB.^39,40^ Importantly, Yagyu *et al.*^40^ suggested that the metal ion is found on the periphery moiety of micelles rather than the core; *i.e.* Pd(ii) ions might be on the surface of the surfactant-complex/organic salt formed by [PdX_4_]^2−^ (X = Br or Cl) and CTA^+^ moieties. With the increase of temperature over time, the dissociative organic salt was reduced and Pd(ii) is slowly released to the aqueous media. This might be the key factor that leads to very monodisperse particles. At the same time, the double bond from oleate can coordinate with Pd(ii) ions, as previously reported by Ghebreyessus *et al.*^41^ It is well known that Pd(ii) complexes are prone to coordinate with alkenes, such as double bonds from olefins. The double bond is rich in electrons and can transfer to contribute to the reduction of Pd(ii). It is also known that unsaturated fatty acids can form free radicals *via* autoxidation but also in the presence of a newly formed Pd(0) surface (acting as heterogeneous catalyst), and also contribute to catalyse the reduction of Pd(ii) ions and particle formation *via* epitaxial growth. The formation of Pd(oleate)_2_ might also occur in the course of the reaction but it can have a less pronounced effect on the kinetics of the reaction due to the fact that there exists an excess of Pd compared to oleate, and Pd oleate salt is insoluble in an aqueous medium.

The reduction of Pd(ii) occurs according with the following reaction.PdCl_4_^2−^ + 2e^−^ → Pd + 4Cl^−^ (*E*_red_ = 0.591 *vs.* SCE)

As described by Kitaguchi *et al.*,^42^ the oxidation potential of oleate is *E*_ox_ = 2.03 V (*E*_red_ = −2.03 V *vs.* SCE). Given the much lower reduction potential of Pd(ii) compared to oleate, it is obvious to rule out that the reduction of Pd is favoured upon oxidation of oleate. The initial seeds of Pd can be generated by reduction of Pd ions and could further grow with the increase of temperature. As an inorganic ion, the chloride ion does not have remarkable stabilization and a selective effect on adsorption, which leads to the formation of an isotropic structure. Therefore, Pd isotropic particles are the most favoured product.^39^

### 2.2 Quantitative evaluation of reduction kinetics by UV-Vis spectroscopy

**Fig. 2:**
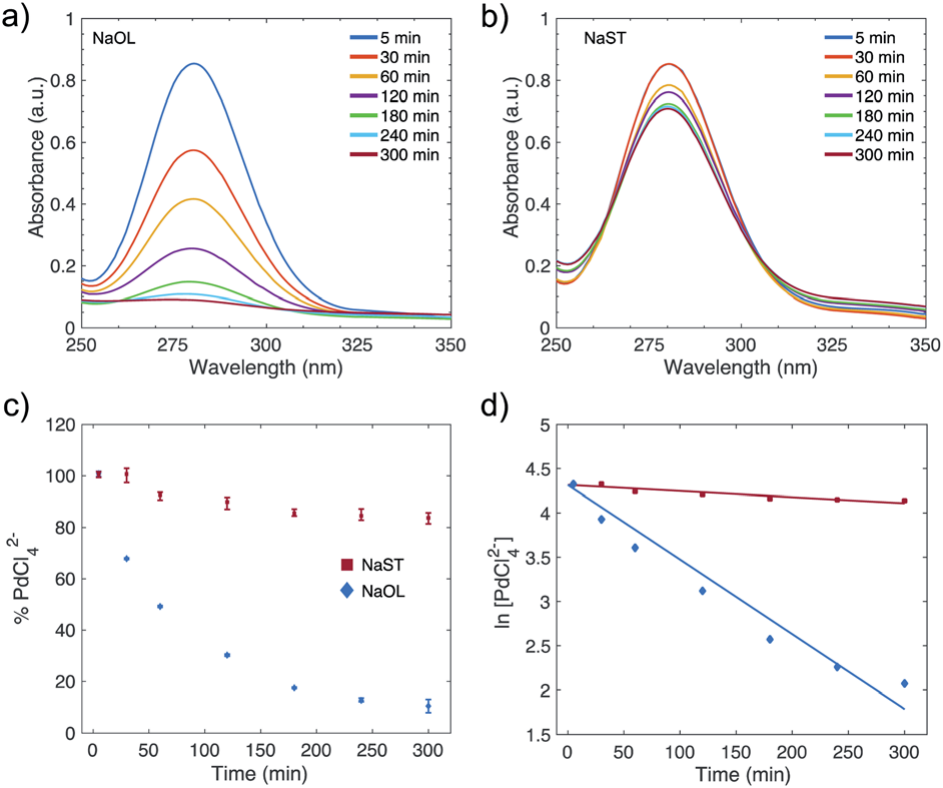
Analysis of the reduction kinetics in the synthesis of Pd NPs. UV-Vis spectra of PdCl_4_^2−^ in the reaction solution after 5, 30, 60, 120, 180, 240, and 300 min for Pd NPs stabilized with (a) NaOL and CTAC, and (b) NaST and CTAC. The spectra were constructed by taking the average of 3 replicate samples. (c) A plot showing the percentage of PdCl_4_^2−^ remaining in the reaction solutions for Pd NPs synthesized with NaOL and CTAC, and with NaST and CTAC, with error bars, measured from the absorbance peak at 280 nm as a function of time. (d) Plots of ln PdCl_4_^2−^ over time, showing the pseudo-first order reaction kinetics involved in the synthesis of Pd NPs stabilized with NaOL and CTAC, and with NaST and CTAC, respectively.

To quantitatively evaluate the reduction kinetics of the Pd-precursor (PdCl_4_^2−^) in the synthesis of Pd NPs, UV-Vis spectroscopy was used. Pd NPs stabilized with NaOL-CTAC and NaST-CTAC were studied. Samples were analyzed at different time intervals of the synthesis; 5, 30, 60, 120, 180, 240 and 300 min. The PdCl_4_^2−^ complex shows two characteristic peaks of 222 nm and 280 nm, which are in accordance with the values presented in the literature.^43^ In this study the reduction kinetics was monitored by following the diminishing absorbance peak at 280 nm. It can be seen in the UV-Vis spectra of PdCl_4_^2−^ that Pd NPs stabilized with NaOL and CTAC (Fig. 2a) show a faster reduction rate compared to the Pd NPs stabilized with NaST and CTAC (Fig. 2b). The spectra are averaged from three replicate samples.

A calibration curve was constructed to calculate the amount of PdCl_4_^2−^ remaining in the reaction solutions and follow the reduction over time. Absorbance was measured at 280 nm from a set of H_2_PdCl_4_ solutions with a known concentration (Fig. S9†). From the calibration curve the percentage of PdCl_4_^2−^ can then be calculated from the amount of Pd(ii) that had been converted to Pd(0). The plot in Fig. 2c shows how the % PdCl_4_^2−^ varies over time in the reaction for the systems with NaOL and CTAC (blue diamonds) and with NaST and CTAC (red squares). After 1 hour of reaction, 50.8% of PdCl_4_^2−^ is reduced for the NaOL and CTAC system, which increases to 87.2% at the optimal reaction time of 4 hours. In comparison, for the NaST and CTAC system only 7.4% and 15.4% of PdCl_4_^2−^ are converted to Pd(0) after 1 and 4 hours reaction, respectively. After 6 hours of reaction the conversion further increases to 89.4% for NaOL and CTAC and 16.3% for the NaST and CTAC system. The analysis of the supernatant of Pd NPs prepared in the presence of CTAC/NaOL after centrifugation was conducted by X-ray fluorescence (see Table S3†), which shows a conversion rate of 81% from Pd(ii) to Pd(0). The reduction of Pd(ii) in an acidic medium in the presence of typical reducing agents such as ascorbic acid and sodium borohydride, even at a higher relative concentration of reducing agent, shows lower conversion rates (Table S4†). The quantitative analysis indicates that the reduction of PdCl_4_^2−^ to Pd(0) is faster and more effective for Pd NPs stabilized with NaOL and CTAC compared to using NaST and CTAC.

In solution-based colloidal synthesis of metal NPs, the reduction of the metal precursor is normally performed in the presence of a reductant. The reaction is considered bimolecular since collision and electron transfer between the reductant and the metal precursor occur.^44^ Thus, the chemical kinetics of the reaction can be considered to follow a second order rate law, where the rate of the reaction depends on the concentration of the two reagents. For a bimolecular reaction A + B → product, the rate of the reaction can be written according to the following equation

1 Rate = *k*′·[*A*][*B*]

where *A* is the metal precursor and *B* is the reductant, corresponding to PdCl_4_^2−^ and NaOL or NaST in our study. If the reductant is supplied in excess in relation to the metal precursor, the concentration can be assumed to remain constant throughout the reaction. This assumption shows that the reduction rate can be simplified to a pseudo first-order reaction and the rate law can be written as2
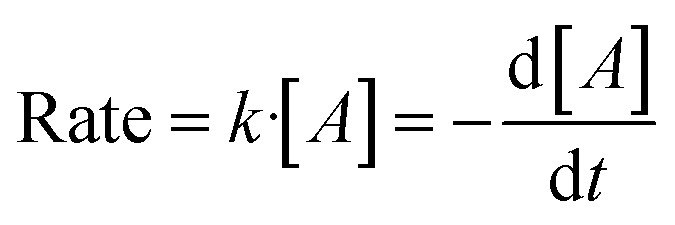


Further integration of eqn (2) gives

3 ln[*A*_*t*_] = −*kt* + ln[*A*_0_]

where *k*= *k*′[*B*] is the rate constant of the reaction, *t* is the reaction time, and [*A*_0_] and [*A*_*t*_] are the concentration PdCl_4_^2−^ at the beginning of the reaction and after a specific reaction time.

The first-order reaction rate law was applied to the Pd NPs stabilized with NaOL and CTAC, and NaST and CTAC (Fig. 2d). The plot of ln[PdCl_4_^2−^] as a function of time decreases linearly for both systems, and thus it can be concluded that they follow a pseudo first-order reaction rate law. From the slope of the lines the *k*-values were calculated to be *k* = 1.27 × 10^−4^ s^−1^ and *k* = 1.17 × 10^−5^ s^−1^ for the NaOL-CTAC and NaST-CTAC systems, respectively. The pseudo first-order model fits well to the data in Fig. 2d, except for the points at 240 and 300 min. The slight deviation at these points may originate from scattering from the Pd NPs in the sample solution which could have contributed to a slight increase in absorbance. A prerequisite for applying the pseudo first-order model to a NP synthesis is that the concentration of the reductant remains constant throughout the synthesis. For our systems, the initial concentration of Pd-precursor PdCl_4_^2−^ is 10.2 mM, and the concentration of the reductant NaOL or NaST was 8 mM and is not present in excess. Nonetheless, it could be argued that NaOL and NaST might reduce several Pd(ii) ions to Pd(0), and therefore the concentration remains constant and would explain why the reaction correlates well with the pseudo first-order reaction.

### 2.3 Nanoparticle characterization

By studying the interactions of NaOL and CTAC with Pd NPs further knowledge of their roles in the synthesis could be elucidated. The zeta potential measured in the stabilizer mixture at different molar ratios showed good colloidal stability under the present conditions (41.3 mV, Table S2†), by the formation of mixed micelles between NaOL and CTAC.^32,45^ To obtain qualitative information about how the stabilizers NaOL and CTAC adsorb onto the surface of the Pd NPs, an FTIR spectrum was recorded. Fig. S10† shows the FTIR spectra of CTAC, NaOL and the Pd NPs. The absorption peaks between 2955 and 2846 cm^−1^ present in all spectra correspond to the symmetric stretching vibrations of methyl and methylene CH_2_ bonds. The peak at 1557 cm^−1^ corresponding to the asymmetric stretching vibration of COO^−^ of NaOL is shifted to 1717 cm^−1^ in Pd NPs, explained by capping of the NP surface. The intense band at 1105 cm^−1^ in the IR spectrum of the Pd NPs can be ascribed to C–N stretching. The bands at 1392 and 1407 cm^−1^ present in the IR spectrum of Pd NPs and CTAC represent the N–CH_3_ symmetric stretching vibrations and show a non-significant shift. Further information about the interaction of the stabilizers with the Pd NPs is obtained from ^1^H NMR spectra of CTAC, NaOL, and the Pd NPs in D_2_O (Fig. S11†). Comparing the Pd NP spectrum with that of CTAC and NaOL, the multiplets at 5.37 ppm are attributed to the olefinic CH in NaOL, and the methylene protons located at 3.38 ppm and between 1.52 and 2.17 are significantly shifted due to the interaction of these capping agents on the surface of the Pd NPs. It can be concluded from the FTIR and ^1^H NMR analysis that CTAC and NaOL are involved in the synthesis of the Pd NPs, by capping and colloidal stabilization of the NP surface.

## 3 Conclusions

This study presents the development of a simple method to synthesize concentrated suspensions of highly monodisperse Pd NPs stabilized with NaOL and CTAC. The Pd NPs were synthesized without using traditional reducing agents, where NaOL acted as a reductant of Pd-precursors. A variety of Pd NP sizes and morphologies could be formed when the temperature, reaction time and type of stabilizers were altered. It was confirmed that the alkyl double bond in NaOL was not the only contributor to the reduction of PdCl_4_^2−^, since Pd NPs could be synthesized with CTAC and the saturated fatty acid salt NaST. Quantitative evaluation of the reduction kinetics using UV-Vis spectroscopy shows that synthesis of Pd NPs stabilized with NaOL and CTAC, and with NaST and CTAC follows pseudo first-order reaction kinetics. The stabilizer combination with NaOL and CTAC provides faster and more effective reduction of Pd-precursors. Qualitative analysis by FTIR and ^1^H NMR shows that NaOL and CTAC adsorb to and stabilize the surface of the Pd NPs. This study presents that in the synthesis of Pd NPs, both fatty acids NaOL and NaST can act as reductants in the absence of traditional reducing agents. We envision that these highly monodisperse and concentrated Pd NP suspensions would show interesting properties in future catalytical or other applications.

## 4 Experimental

### 4.1 Chemicals

Sodium oleate (NaOL) (≥97%, TCI), palladium(ii)chloride (≥99.9%, PdCl_2_, Sigma), hexadecyltrimethylammonium chloride (CTAC) solution (25 wt%, Sigma), hexadecyltrimethylammonium bromide (CTAB) (≥99%, Sigma), hydrochloric acid (HCl) (37%, Sigma), sodium stearate (NaST) (≥99.9%, Sigma), potassium chloride (KCl) (≥99.9%, Sigma), and deuterium oxide (D_2_O) (≥99.9%, Sigma) were used. Deionized (DI) water (MilliQ, 18.2 MΩ) was used in the synthesis experiments. Aqueous H_2_PdCl_4_ solution (50 mM) was prepared by dissolving 177.3 mg PdCl_2_ in 20 mL HCl (100 mM) at room temperature for 3 hours. Samples for NMR measurements were synthesized by replacing all DI water by D_2_O.

### 4.2 Synthesis of Pd nanoparticles

In the synthesis of Pd NPs, reaction conditions were optimized to obtain monodisperse NPs. Synthesis was performed by mixing 3.92 mL CTAC solution (50 mM) with 0.98 mL NaOL solution (50 mM) in a 20 mL glass vial. Then, 1.25 mL H_2_PdCl_4_ solution (50 mM) was added, creating a turbid orange suspension. The vial was capped and was left in a convection oven at 100 °C for 4 hours. The final nanoparticle suspensions were purified by two consecutive cycles of centrifugation (VWR Micro Star 12) at 12 300×*g* for 20 min, cleaned with DI water between cycles and redispersed in the same volume (6.15 mL) of DI water. Different reagent concentrations and parameters were explored for the synthesis of Pd NPs and are listed in Table S1.†

### 4.3 Kinetic study by UV-Vis spectroscopy

To study the reaction kinetics of the Pd NP synthesis, samples were analyzed after specific time intervals by UV-Vis spectroscopy. Samples of the reaction solution (0.15 mL) were taken at specific time points during the reaction; 5, 30, 60, 120, 180, 240, and 300 min, and were added to 5.025 mL of room temperature saturated KCl-solution and were mixed. Aliquots of this stock solution (0.375 mL) were added to 3 Eppendorf tubes containing KCl-solution (1.125 mL in each). Dilution with KCl solution was performed to avoid hydrolysis of the PdCl_4_^2−^ complex and to quench the reaction. The final solutions were centrifuged (VWR Micro Star 12) at 12 300×*g* for 20 min to separate Pd NPs from the PdCl_4_^2−^ complex. The supernatant was analyzed with UV-Vis spectroscopy. To calculate the percentage of PdCl_4_^2−^ remaining in the reaction solutions a standard plot was constructed. This was performed by measuring the absorbance at 280 nm for a series of H_2_PdCl_4_ solutions with a known concentration, in saturated KCl solution (Fig. S9†).

### 4.4 Sample preparation for characterization

Fourier transform infrared spectroscopy (FTIR) was used to study the chemical composition of NaOL, CTAC and the Pd NPs. Samples that were in solution were completely dried prior to analysis. The CTAC solution was dried in an oven at 70 °C for 12 h. The Pd NPs stabilized with NaOL and CTAC were synthesized according to the optimal procedure and were centrifuged once (VWR Micro Star 12) at 12 300×*g* for 20 min. The resulting supernatant was discarded, and the concentrated Pd NP pellet was dried in an oven at 80 °C for 5 h. Remaining water in the CTAC and Pd NP samples was removed using a vacuum oven at 40 °C for 20 h. The interaction of NaOL, CTAC and the Pd NPs was studied by nuclear magnetic resonance (NMR) spectroscopy. CTAC was dried as explained in the FTIR section. NaOL and dried CTAC were dissolved in D_2_O. Pd NPs stabilized with NaOL and CTAC were synthesized according to the optimal method, except that DI water was replaced with D_2_O. The freshly synthesized NP suspension and the reagent solutions were added to NMR-tubes.

The analysis of the conversion efficiency of Pd(ii) to Pd(0) was conducted by the comparative assessment of the Pd nanoparticle concentration in the pristine suspension and the remaining Pd ions available in the suspension supernatant after ultracentrifugation. We used ultracentrifugation to ensure that most of the particles Pd(0) were sedimented at the bottom of the tube and only Pd(ii) or tiny clusters are available in the suspension supernatant. The centrifugation was conducted by placing 8 mL of Pd nanoparticle suspension in a screw-cap sealed polycarbonate tube and centrifuging at 30 000 rpm for 30 min using an ultracentrifuge BeckmanCoulter Optima XL-100K. The concentration of palladium was evaluated in both the pristine suspension and suspension supernatant by X-ray fluorescence (Panalytical Axios). For this purpose, a standard curve (kcp *versus* ppm) was built by using Pd standard solutions as shown in Table S4.†

### 4.5 Characterization

The nanoparticle morphology and size distribution were investigated using a transmission electron microscope (TEM) FEI Tecnai T20 operating at 200 kV. The study of the crystal structure of the NPs was conducted by high-resolution transmission electron microscopy (HRTEM) imaging and selected area electron diffraction (SAED) performed with a FEI XFEG TITAN electron microscope operating at 300 kV. Samples for TEM-analysis were prepared by drop casting concentrated nanoparticle suspensions onto a carbon coated Cu TEM-grid. To calculate the particle size distribution, size measurements were performed on >200 Pd NPs in each sample. The absorbance spectra and reduction kinetics study of the Pd-precursor was conducted using an Agilent Cary 60 ultraviolet-visible (UV-Vis) spectrophotometer with a xenon flash lamp (80 Hz) as a light source. The chemical composition of the reagents and Pd NPs was studied with a Perkin Elmer FTIR spectrometer using a diamond attenuated total reflectance infrared (ATR-IR) crystal, and using a NMR spectrometer operating at 400 Hz.

## Conflicts of interest

Anna Pekkari is currently an employee of AstraZeneca.

## Supplementary Material

NA-003-D1NA00052G-s001
