# Supplementary material for: Synthesis of highly monodisperse Pd nanoparticles using a binary surfactant combination and sodium oleate as a reductant
Source: Nanoscale Adv. 2021 Mar 5;3(9):2481–7. doi: 10.1039/d1na00052g (PMC9417948; doi:10.1039/d1na00052g)
Supplement: NA-003-D1NA00052G-s001 [file NA-003-D1NA00052G-s001.pdf]

Electronic Supplementary Material (ESI) for Nanoscale Advances.
This journal is © The Royal Society of Chemistry 2021

Supporting Information

Synthesis of highly monodisperse Pd nanoparticles using a binary surfactant combination and sodium oleate as reductant

Anna Pekkari[1], Xin Wen[1], Jessica Orrego-Hernández[1], Robson Rosa da Silva[1], Shun Kondo[2], Eva Olsson[2], Hanna Härelind[1], and Kasper Moth-Poulsen[1]*

[1]Applied Chemistry, Department of Chemistry and Chemical Engineering, Chalmers University of Technology, 41296 Gothenburg, Sweden

[2]Nano and Biophysics, Department of Physics, Chalmers University of Technology, 41296 Gothenburg, Sweden

Email: kasper.moth-poulsen@chalmers.se

Experimental parameters

In Table S1, the experimental parameters that were used in the synthesis of the Pd NPs are listed.

Table S1. Experimental parameters for the synthesis of Pd NPs.

Sample	T (℃)	reaction time (h)	heating	c Pd (mM)	c NaOL (mM)	c NaST (mM)	c CTAC (mM)	c CTAB (mM)	Figure
1	100	4	oven	10.2	8	-	31.9	-	1
2	100	1	oven	10.2	8	-	31.9	-	S1a
3	100	2	oven	10.2	8	-	31.9	-	S1b
4	50	4	oven	10.2	8	-	31.9	-	S2a
5	80	4	oven	10.2	8	-	31.9	-	S2b
6	100	2	oil bath	10.2	8	-	31.9	-	S3a
7	100	4	oil bath	10.2	8	-	31.9	-	S3b
8	100	4	oven	10.2	8	-	-	31.9	S4
9	100	4	oil bath	10.2	8	-	-	-	S6
10	100	4	oil bath	10.2	15.9	-	-	-	S6
11	100	4	oil bath	10.2	39.8	-	-	-	S6
12	10	4	oven	10.2	-	8	31.9	-	S5
13	100	4	oven	10.2	-	8	-	-	S7

Zeta potential measurements were performed on solutions of NaOL and CTAC at different molar ratios (Table S2) to investigate the colloidal stability. The present reaction conditions are α_{CTAC}= 0.8 showing good colloidal stability.

Table S2. Values of the Zeta-potential for NaOL and CTAC solutions at different molar ratios, measured at 25 ºC. The values are an average of three or more measurements. Where α_{CTAC} = nCTAC/ (nCTAC+ nNaOL).

α_{CTAC}	Zeta potential (mV)	Standard deviation (mV)
1	69.9	15.4
0.9	52.1	10.9
0.8	41.3	19
0.5	37.3	1.7
0	-54.0	17.1

Table S3. Literature review showing the reduction efficiency of Pd(II) in the presence of traditional reducing agents and acidic medium.

Pd source	Reducing agent	Pd:[R] molar ratio	Acidic medium	%Conversion	Ref.
$[PdCl_4]^{2-}$	Ascorbic Acid	1:5.5	$[HNO_3]$ 0.5 M	≈ 30 %	[1]
$[PdCl_4]^{2-}$	Ascorbic Acid	1:3	[HCl] < 0.3 M	≈100 %	[2]
			[HCl] > 0.5 M	<5 %	[2]
$[PdCl_4]^{2-}$	Sodium borohydride	1:3	[HCl] 0-3 M	≈ 100 %	[2]
			$[H_2SO_4]$ 0-3 M	≈ 100 %	[2]
$[PdCl_4]^{2-}$	Sodium Phosphinate	1:3	[HCl] 0-3 M	≈ 100 %	[2]
			$[H_2SO_4]$ 0-3 M	≈ 100 %	[2]
[NaOL)	Sodium oleate	1:0.78	Aqueous acid solution (pH=1.97)	≈ 81 %	Our work

[R]= reducing agent

Table S4. Calibration curve based on X-ray fluorescence (XRF) analysis of Pd standard solutions. The x axis refers to concentration of Pd in particles per million (ppm) and the y axis refers to counts (kcps) from XRF detector for Pd Kα1 peak (21.18 KeV).

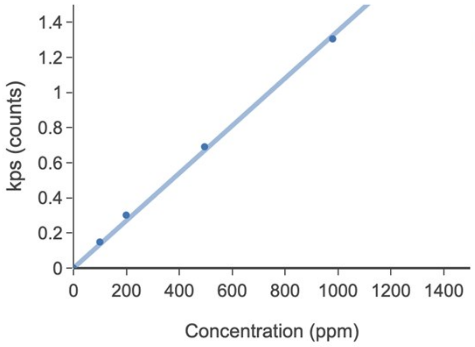

	Concentration (ppm)	Standard deviation (ppm)
Pristine Pd suspension	1320	40
Supernatant	260.2	6.5

High resolution scanning transmission electron microscopy (HRSTEM) imaging of Pd nanoparticles

Through the observation in the TEM, it is discovered that most of the Pd NPs are polycrystals and only several Pd NPs are single- crystals. A HRSTEM image of a single-crystal Pd NP is shown in Figure S1 (a). It can be seen that there are stacking faults and twins in the single-crystal Pd NPs. The {111} and {200} lattice planes and their interplanar spacings are marked in the HRSTEM image (Figure S1 (a)). Figure S1 (b) shows a HRTEM image of a single-crystal Pd NP and its corresponding FFT pattern is displayed in Figure S1 (c), indicating that the zone axis of this crystal unit is [-112]. The spots resulted from (220), (311) and (1-11) lattice planes are marked in Figure S1 (c).

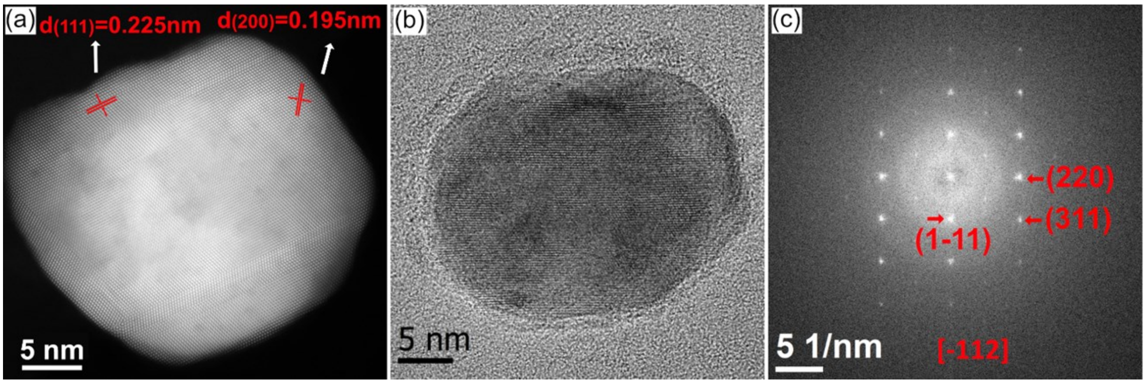

Figure S1. (a) High resolution scanning transmission electron microscopy (HRSTEM) image of a single-crystal Pd NP containing stacking faults and twin planes. The {111} and {200} lattice planes, marked in the image by a pair of parallel red lines, have an interplanar spacing of 0.225 nm and 0.195 nm, respectively. (b) HRTEM image of a single-crystal Pd NP. (c) Corresponding FFT pattern of (b), indicating that the zone axis of this crystal unit is [-112]. The spots resulted from (220), (311) and (1-11) lattice planes are pointed by red arrows.

Synthesis optimization of Pd NPs

For the development of the Pd NP synthesis a thorough evaluation of reaction parameters and the influence on the particle size and morphology was performed.

Reaction time

Optimization of the reaction time was performed by synthesizing Pd NPs for different times. **Figure S2**a and **S2**b shows a TEM image after 1h and 2h reaction respectively.

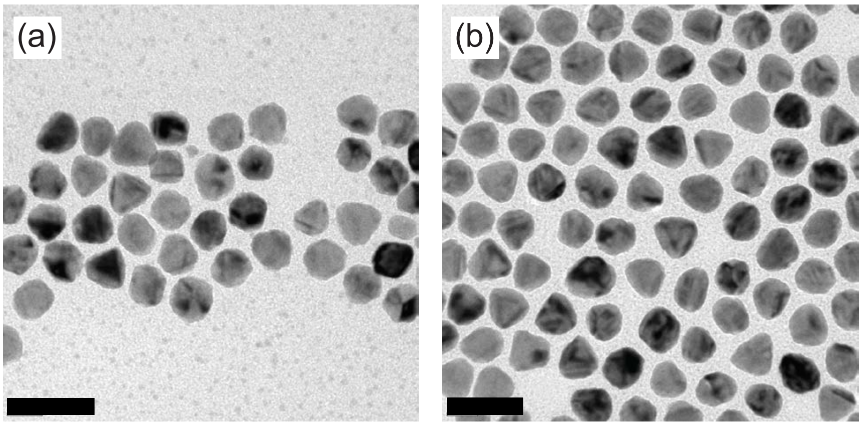

Figure S2. TEM images of Pd NPs synthesized in an oven at 100 °C. a) 1h reaction gave homogeneous Pd NPs with plenty of particle seeds are present. b) After 2h synthesis Pd NPs are homogeneous and few seeds are present. Scale bars are 50 nm.

Temperature

The synthesis was performed at different temperatures to evaluate the effect on the formed Pd NPs. At 50 °C the Pd-complex could not be dissolved and it appeared as an orange suspension (Figure S3a), whereas at 80 °C Pd NPs formed (Figure S3b).

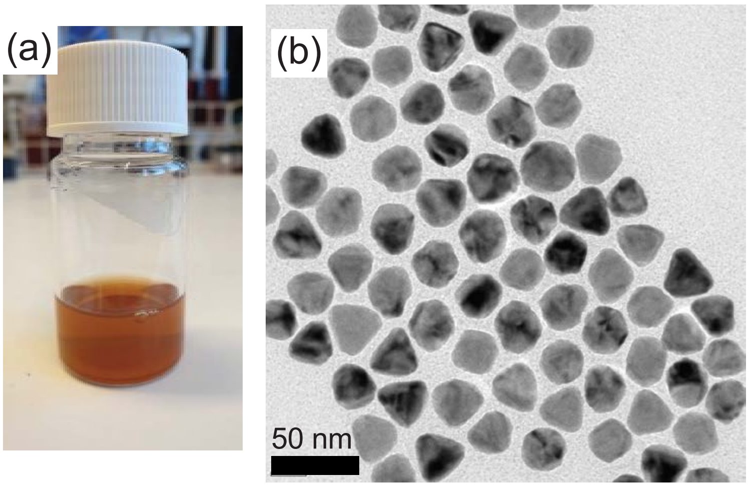

Figure S3. a) Image of the reaction solution after 4h at 50 °C that shows the insoluble Pd-complex. b) TEM image of Pd NPs prepared at 4h at 80 °C, that are uniform in size.

Synthesis in oil bath

The Pd NPs were synthesized in an oil bath at 100 °C. Pd NPs show reproducible shapes and morphologies compared to the optimal synthesis conditions when synthesized for 2h (Figure S4a) and 4h (Figure S4b).

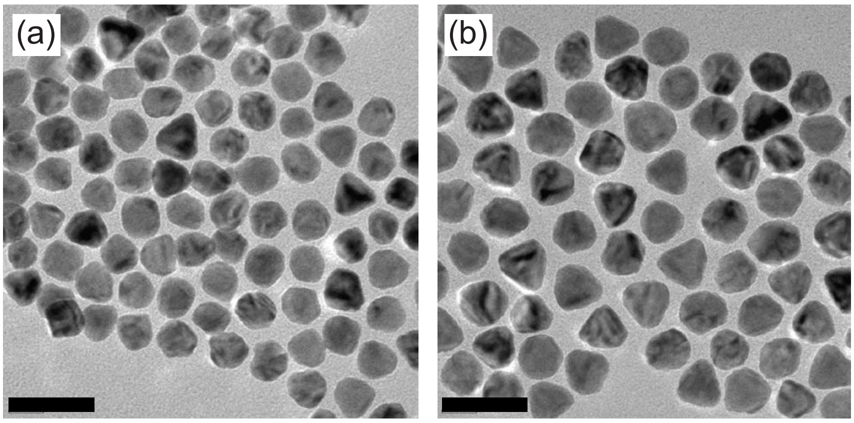

Figure S4. TEM images of Pd NPs synthesized in an oil bath at 100 °C. a) After 2 h synthesis uniform Pd NP are formed. b) After 4 h Pd NPs are uniform with slightly larger size. Scale bars are 50 nm.

Pd NPs stabilized with NaOL and CTAB

The effect on the shapes of Pd NPs when replacing CTAC with CTAB in the stabilizer mixture is shown in Figure S5. The shapes of Pd NPs consist of a mixture of cubes, bars and “arrows”.

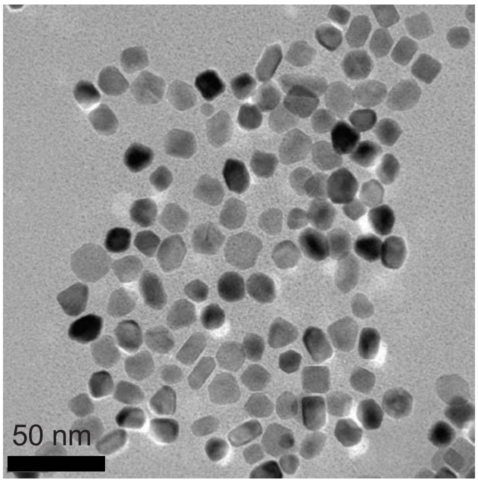

Figure S5. TEM image of Pd NPs prepared according to the optimal reaction conditions except using CTAB and NaOL as stabilizers.

Pd NPs stabilized with NaST and CTAC

Pd NP were synthesized with NaST and CTAC as stabilizers. The shapes are a mixture of triangles, twinned and polycrystalline Pd NPs (Figure S6).

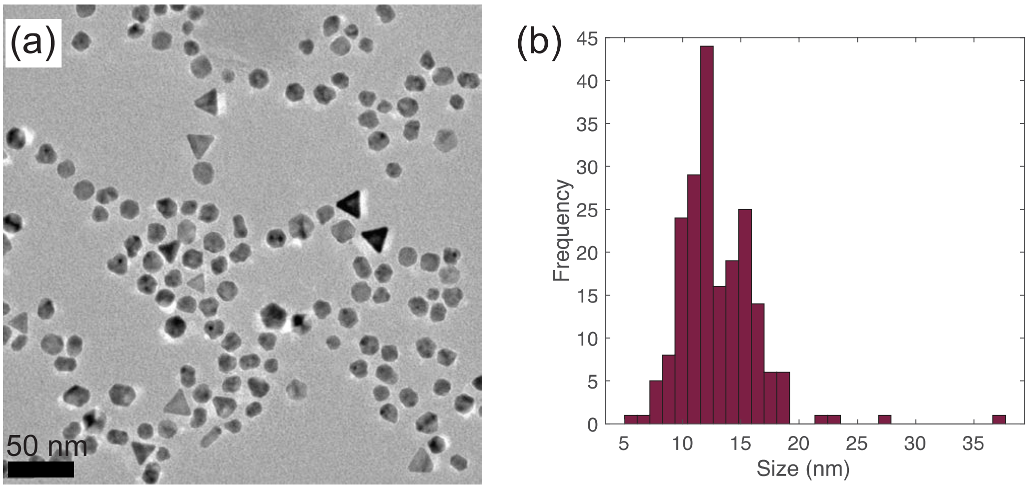

Figure S6. a) TEM image of Pd NPs prepared with optimal reaction conditions except using NaST and CTAC as stabilizers. b) Histogram of size distribution of Pd NPs with average size of 13 nm ± 19 %.

Pd NPs stabilized with NaOL

Pd NPs stabilized with NaOL were synthesized in an oil bath (Figure S7). The NPs were stabilized with different concentrations of NaOL and showed poor colloidal stability and visual precipitation (Figure S7a). TEM image of the small Pd NPs synthesized with 39.8 mM NaOL (Figure S7b).

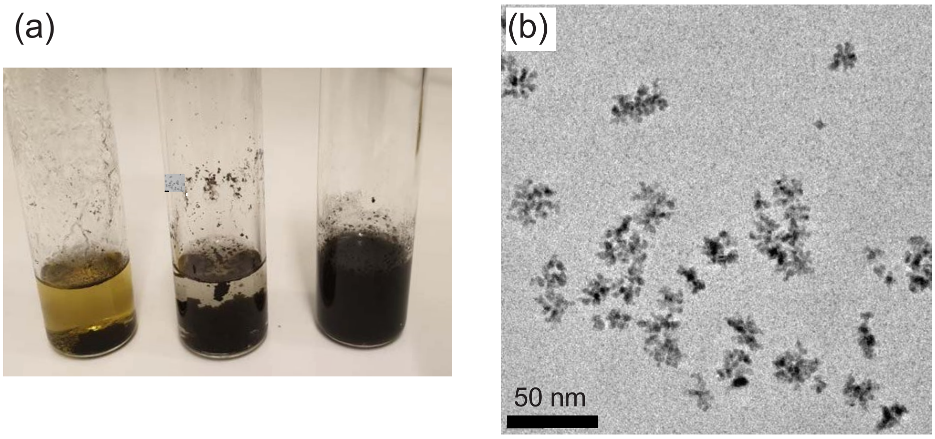

Figure S7. a) NaOL-stabilized Pd NP suspensions after 1h, at 100 °C in an oil bath. The Pd NPs were stabilized with different concentrations of NaOL; 8 mM (left), 15.9 mM (middle), and 39.8 mM (right). b) TEM image of Pd NP stabilized with 39.8 mM NaOL.

Pd NPs stabilized with NaST

For evaluation of the reduction mechanisms in the formation of Pd NPs NaOL was replaced by NaST. The initial precursor suspension with 8 mM NaST is turbid (Figure S8a), after 2h in oven a black precipitate is formed at the surface of the orange solution (Figure S8b). TEM

image of Pd NPs after 4h reaction, sampled from the black precipitate at the surface of the solution shows Pd NPs and the presence of many particle seeds (Figure S8c).

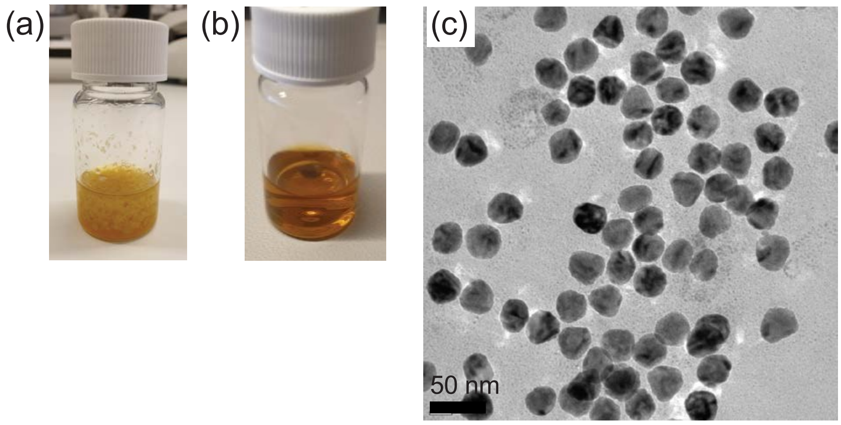

Figure S8. Pd reaction mixtures stabilized with 8 mM NaST. a) Initial insoluble mixture, b) the mixture after 2h reaction. c) TEM image of Pd NPs stabilized with 8 mM NaST after 4h reaction.

Nanoparticle characterization

Standard plot for quantitative evaluation of reduction kinetics

To calculate the concentration of $PdCl_4^{2-}$ remaining in the reaction solution, a standard plot was constructed. The absorbance spectra for $PdCl_4^{2-}$ solutions in saturated KCl solution with different concentration (1, 10, 25, 50, 75, and 100 μM), is shown in Figure S9a. The standard plot showing the absorbance at 280 nm as a function of $PdCl_4^{2-}$ concentration is shown in Figure S9b.

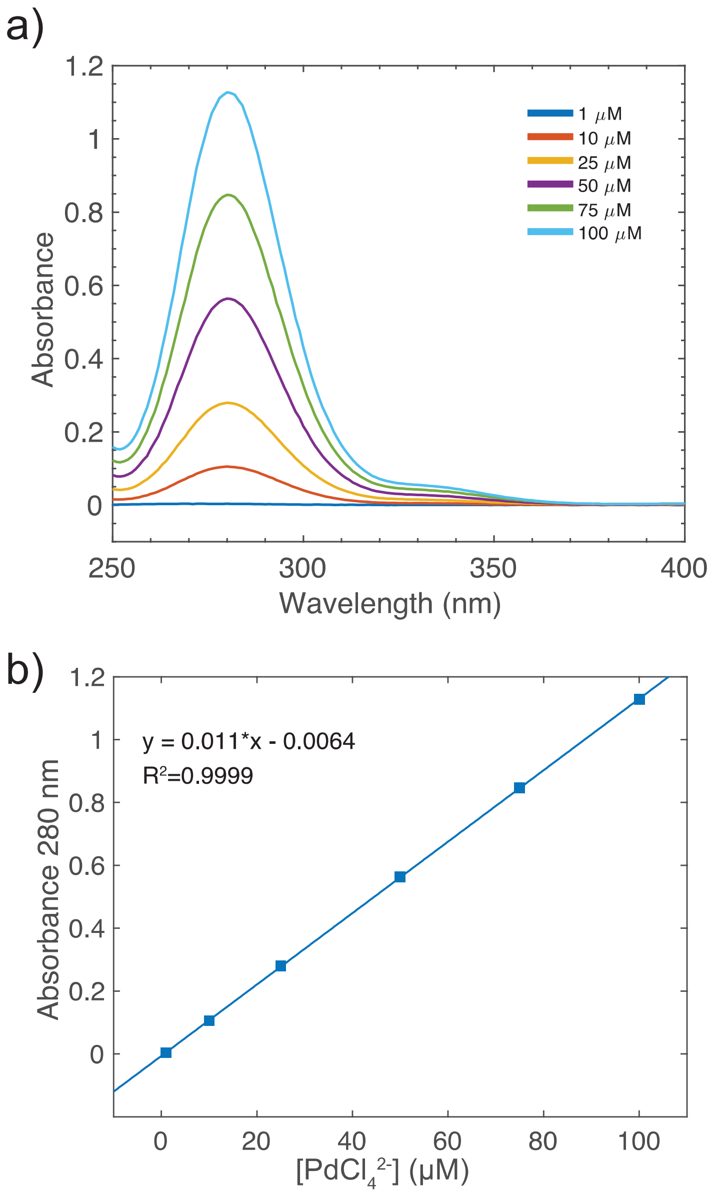

Figure S9. a) UV-VIS absorbance spectra of standard $PdCl_4^{2-}$ solutions in KCl. b) Standard plot showing the absorbance at 280 nm as a function of the $PdCl_4^{2-}$ concentration.

Qualitative characterization using FTIR and ^{1}H NMR

The Pd NPs and the stabilizers CTAC and NaOL were analyzed with FTIR (Figure S10) and with ^{1}H NMR in D_2O (Figure S11)

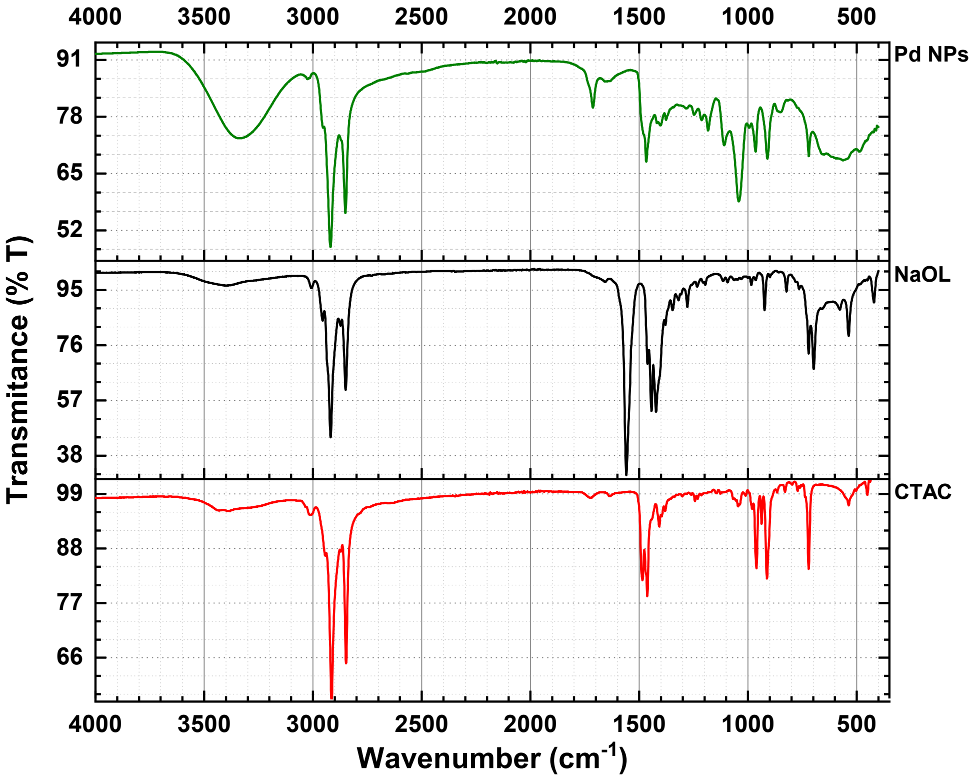

Figure S10. FTIR spectra of the reagents used in the synthesis, and of Pd NPs. From bottom to top; CTAC (red), NaOL (black), and Pd NPs (green).

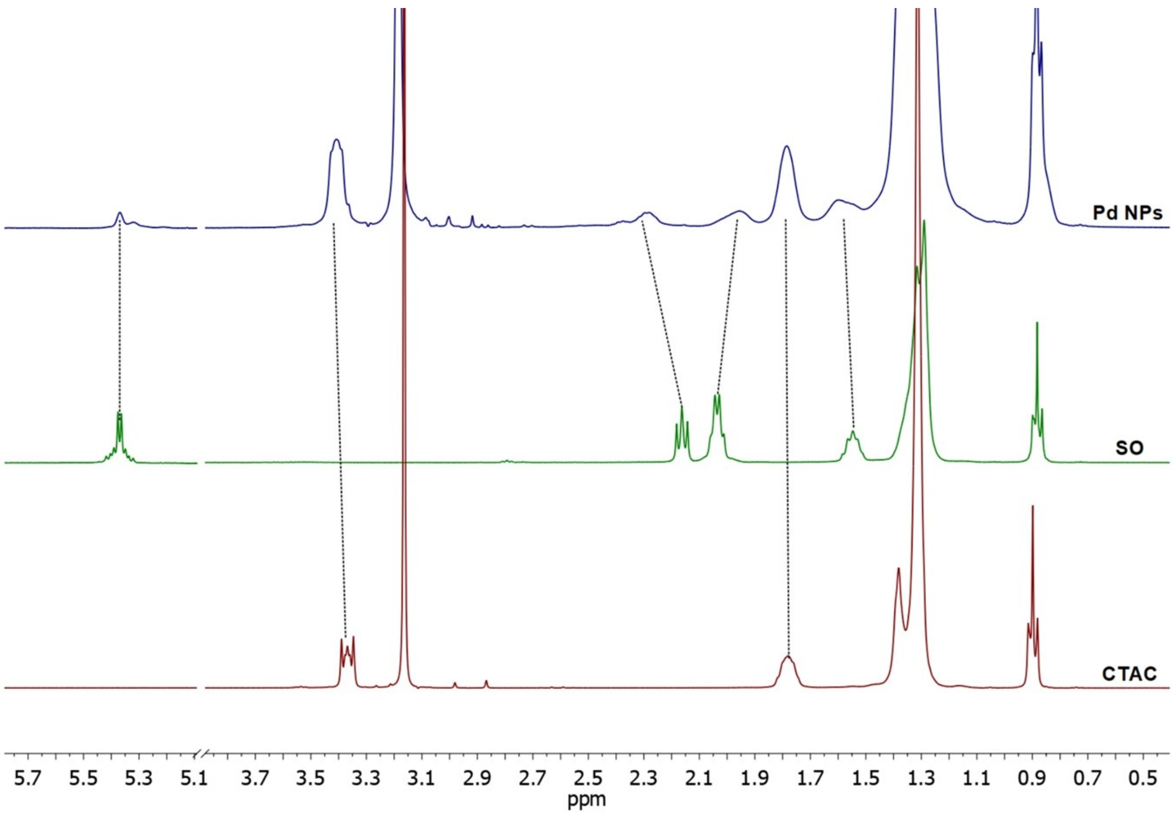

Figure S11. ^{1}H NMR (D_2O) spectra of CTAC (bottom), NaOL (middle) and the Pd NPs (top).
